# Pipeline design to identify key features and classify the chemotherapy response on lung cancer patients using large-scale genetic data

**DOI:** 10.1186/s12918-018-0615-5

**Published:** 2018-11-20

**Authors:** María Gabriela Valdés, Iván Galván-Femenía, Vicent Ribas Ripoll, Xavier Duran, Jun Yokota, Ricard Gavaldà, Xavier Rafael-Palou, Rafael de Cid

**Affiliations:** 1 Eurecat. Technology Centre of Catalonia, Av. Diagonal 177, 9th floor, Barcelona, 08018 Spain; 2 PMPPC-IGTP. Programa de Medicina Predictiva i Personalitzada del Càncer - Institut Germans Trias i Pujol (IGTP). Genomes for Life - GCAT lab Group, Badalona, Spain; 3 PMPPC-IGTP. Programa de Medicina Predictiva i Personalitzada del Càncer - Institut Germans Trias i Pujol (IGTP). CancerGenome Biology, Badalona, Spain; 4 Universitat Politècnica de Catalunya, Barcelona, Spain; 5 Barcelona Graduate School of Mathematics, BGSMath, Barcelona, Spain

**Keywords:** GWAS, Machine learning, Classification, Feature selection, Lung cancer

## Abstract

**Background:**

During the last decade, the interest to apply machine learning algorithms to genomic data has increased in many bioinformatics applications. Analyzing this type of data entails difficulties for managing high-dimensional data, class imbalance for knowledge extraction, identifying important features and classifying individuals. In this study, we propose a general framework to tackle these challenges with different machine learning algorithms and techniques. We apply the configuration of this framework on lung cancer patients, identifying genetic signatures for classifying response to drug treatment response. We intersect these relevant SNPs with the GWAS Catalog of the National Human Genome Research Institute and explore the Regulomedb, GTEx databases for functional analysis purposes.

**Results:**

The machine learning based solution proposed in this study is a scalable and flexible alternative to the classical uni-variate regression approach to analyze large-scale data. From 36 experiments executed using the machine learning framework design, we obtain good classification performance from the top 5 models with the highest cross-validation score and the smallest standard deviation. One thousand two hundred twenty four SNPs corresponding to the key features from the top 20 models (cross validation F1 mean >= 0.65) were compared with the GWAS Catalog finding no intersection with genome-wide significant reported hits. From these, new genetic signatures in *MAE*, *CEP104*, *PRKCZ* and *ADRB2* show relevant biological regulatory functionality related to lung physiology.

**Conclusions:**

We have defined a machine learning framework using data with an unbalanced large data-set of SNP-arrays and imputed genotyping data from a pharmacogenomics study in lung cancer patients subjected to first-line platinum-based treatment. This approach found genome signals with no genome-wide significance in the uni-variate regression approach (GWAS Catalog) that are valuable for classifying patients, only few of them with related biological function. The effect results of these variants can be explained by the recently proposed omnigenic model hypothesis, which states that complex traits can be influenced mostly by genes outside not only by the “core genes”, mainly found by the genome-wide significant SNPs, but also by the rest of genes outside of the “core pathways” with apparent unrelated biological functionality.

**Electronic supplementary material:**

The online version of this article (10.1186/s12918-018-0615-5) contains supplementary material, which is available to authorized users.

## Background

All human diseases are influenced to some extent by genitic variability, and yet much of these genetic consequences are still not fully characterized [1]. The heritability of a trait or disease is defined as the fraction of phenotypic variability attributable to genetic variation [2]. First studies done by medical geneticists were focused on single-gene disorders, which result from mutations in a single gene and as a result, any individual with a mutant allele of this gene has the disease with 100% chance. Whenever the latter case occurs, such genetic effect is called highly penetrant. This type of disorders tend to be uncommon. When the percentage of penetrance is lower, there are individuals who have the predisposing genotype, but do not develop the disease. This happens when other genes play a role in the determination of the disease, or also because of environmental effects. This kind of diseases are called multi-factorial or complex inheritance disorders. Multi-factorial disorders have much higher frequencies in the population and have reduced heritability rates.

Initial approaches mimicking Mendelian approaches, looking for driver genes of the diseases, consisted of analyzing a group of prior “candidate genes” and their effect to a certain trait. Other studies were based on family-based linkage, analyzing inheritance patterns in thousands of genomic markers. In 2003 the genome-wide association (GWA) method appeared as a promise to identify many of the genes involved in complex diseases. In these GWA studies (GWAS), hundreds of thousands of (mainly) single nucleotide polymorphisms (SNPs) are analyzed without priors. If GWAS is used as a case-control study, it is based on a comparison of allele frequencies between groups of affected and unaffected individuals from a population. A particular allele (the variant form of a given gene) is said to be associated with the trait (risk allele) if it occurs at a significantly higher frequency among affected individuals as compared with those in the control group. This strategy has been applied with success to identify hundreds of variants (reviewed in Yang et al. 2017) [3].

The GWAS’s underlying rationale is the “common disease, common variant” hypothesis, referring to the fact that common diseases are attributable in part to allelic variants present in more that 1–5% of the population [4]. But even though these studies have identified hundreds of genetic variants and genes linked to a trait, providing valuable insights into their complexity, both the individual and cumulative effects of these variants have been disappointingly small and very far of explaining the heritability estimates of these traits. This arises as the problem of “missing heritability”. Many hypothesis have been suggested to explain this missing heritability in complex diseases; univariate statistical tests used in GWAS include statistical corrections that lead to very few of the initial variables, low power to detect gene-gene interactions (epistasis), lack of environment consideration, epigenomics, among others [4, 5].

There are still many doubts revolving around missing heritability. This has been an important question to solve, because understanding the genetic variations contribution to these common conditions may contribute to better prevention, diagnosis and treatment in a large part of the population.

A common alternative of methodological approximation to tackle the missing heritability problem, that is the inter-individual variance explained by genetic factors (i.e. variants) not explained so far, is to use machine learning (ML) methods to discover epistatic and non-epistatic polygenic effects in complex diseases [6].

In genomic medicine, random forest (RF) methods have shown to be able to select several genomic regions of interest without substantially increasing the number of false-positive signals compared to the most conservative candidate-gene approach (Bureau et al. 2003). Nowadays numerous ML algorithms (RF, k nearest neighbors (KNN), support vector machine (SVM), etc.) are currently used in biomedical science [7–9] in genome-wide approaches, and its application will rise since floods of multidimensional data are coming with electronic health record (EHR) data accessibility and low cost omics data generation (e.g. NextSeq data, mebalomome).

Lung cancer is the most common cancer in the world, and the leading cause of mortality among cancer-related deaths. Cancer and treatment response is clearly modified by inherited factors, and there is a major interest of developing customized treatments based on patients profiling. The Non-Small-Cell-Lung-Cancer (NSCLC), being the most common form, has an overall 5-years survival of less than 15% [10]. NSCLC is a histological diverse group of tumors, with major classes being squamous (SCC), adenocarcinoma (ADC), and large cell carcinoma (LCC), and commonly, all these tumors have been treated homogeneously with cytotoxic chemotherapy treatment [11]. Attempts to develop more precise treatments has been established by genome-wide studies (GWAS), used to identify predisposition and prognostic biomarkers [12–17].

In precision medicine, ML is used for molecular diagnosis in liquid biopsies to define robust signatures for specific states [18], as well as on disease management of chronic disorders, as Diabetes mellitus Diabetes mellitus (DM). DM is a dynamic field where data integration motivates its application in multiple domains, with good predictive scores (SVM accuracy = 81.3%, RF AUC = 0.80) in [19]. In cancer, another field of interest, ML algorithms has been used for defining prognostic models in Lung cancer patients based on clinical variables [20], and also including genomic profiling in other forms of cancer [21].

The accuracy and the predictive ability of ML algorithms depends of the data, as well the outcome analyzed. Furthermore algorithms should be applied in a sufficiently large dataset for the algorithm to be trained appropriately, and extract high quality of knowledge. However, this is a problem in clinical datasets where the number of patients are small, and contains a rich dataset of variables to be analyzed. In this sense to gain insight in the knowledge as well as improving predictive models, our strategy is to maximize the discovery and validation phase trough unbalanced and heterogeneous data, through the combination of several algorithms with the minimum computational cost.

Here we present a framework based on a pipeline of ML-based steps, developed in a centralized environment (i.e. using a single node, taking advantage of multi-core architecture and parallel library implementations). We implemented the pipeline in a large-scale genetic data-set of lung cancer (LC) of small number of patients to define prognostic models of survival according to the outcome to first-line platinum-based treatment, and gain insight in genetic variability of treatment response.

## Methods

### Cancer data-set

The data-set includes genome-wide data from a pharmacogenomics study in patients with advanced NSCLC [22] subjected to first-line platinum-based treatment. As the main outcome, we considered the survival response, classified under clinical evaluation on the RECIST criteria (response evaluation criteria in solid tumors) as Non responders (DP, Disease progression) and Responders (PR, CR, SD, partial/complete response and stable disease). Responders and non responders to treatment were labeled as class 0 (137 patients) and 1 (41 patients) respectively. The following relevant clinical and socio-demographic variables were included in the analysis and are described elsewhere [23] (Table 1): gender (Male: 0.78, Female: 0.22), smoker (Yes: 0.94 No: 0.06), histology (adenocarcinoma: 0.56, squamos cell carcinoma: 0.36, large cell carcinoma: 0.05, others: 0.03), the ECOG (Eastern Cooperative Oncology Group) Scale of Performance Status (0: 0.33, 1: 0.64, 2: 0.01, NA: 0.01), arm (control arm: 0.53, biomarker-directed arm 0.47), chemotherapy treatment (docetaxel/cisplatin: 0.69, gemcitabine/cisplatin: 0.25, docetaxel: 0.06).

**Table 1:** Relevant clinical and socio-demographic variables in the ML-based analysis

		BREC		Disease progression			
				No		Yes	
		N	%	N	%	N	%
Gender							
	Male (1)	139	78	104	76	35	85
	Female (2)	39	22	33	24	6	15
Smoker							
	Yes (1)	167	94	126	92	41	100
	No (2)	10	6	10	7	0	0
	NA	1	0	1	1	0	0
ECOG							
	0	59	33	45	33	14	34
	1	114	64	88	65	26	64
	2	2	1	2	1	0	0
	NA	3	2	2	1	1	2
Histology							
	ADCA (1)	99	56	83	61	16	39
	SCC (2)	64	36	44	32	20	49
	LCC (3)	6	3	6	4	0	0
	Others (4)	9	5	4	3	5	12
Treatment							
	doce/cis (1)	123	69	93	68	30	73
	gemci/cis (2)	44	25	36	26	8	20
	doce (3)	11	6	8	6	3	7
Arm							
	Control	95	53	72	53	23	56
	Biomarker-directed	83	47	65	47	18	44
RECIST							
	PD (1)	41	23				
	SD (0)	56	31				
	PR (0)	58	32				
	CR (0)	23	14				

Genome-wide genotypes were generated with SNP-array technology using the Infinium HTS Assay, HumanCoreExome-24v1-0 BeadChip, (ILLUMINA, San Diego, CA), and later imputed (SHAPEIT [24], IMPUTE2 [25]), to generate a data-set of 24.873.940 SNPs [22], from which 8.717.047 SNPs from autosomal chromosomes were retained for the association analysis (imputation score > 0.7, MAF > 0.01, LD < 0.2).

For ML approaches we transform genotypes (pair of G, A, C, T) to numerical codes, where each genotype is encoded as a single numeric feature that reflects the number of minor alleles. Homozygous major, heterozygous and homozygous minor are encoded as 0, 1 and 2, assuming an additive effect of the derived allele encoded gene products. This results in a minimal number of generated features while preserving all information. To facilitate ML exploration, for inheritance modelling, in this study we only consider the additive model (0, 1, 2) since it has been shown to capture most of the genetic effects [26].

### Pipeline configuration

The pipeline configuration is the core of the framework applied in this study. It was designed to deal with the difficulties that arise from the nature of the SNP data and our objectives: presence of missing values, different measurement units (features coming with heterogeneous format), high dimensionality, small number of samples, presence of class imbalance, identify key features and need to classify according to response to treatment of LC.

**Fig. 1:**
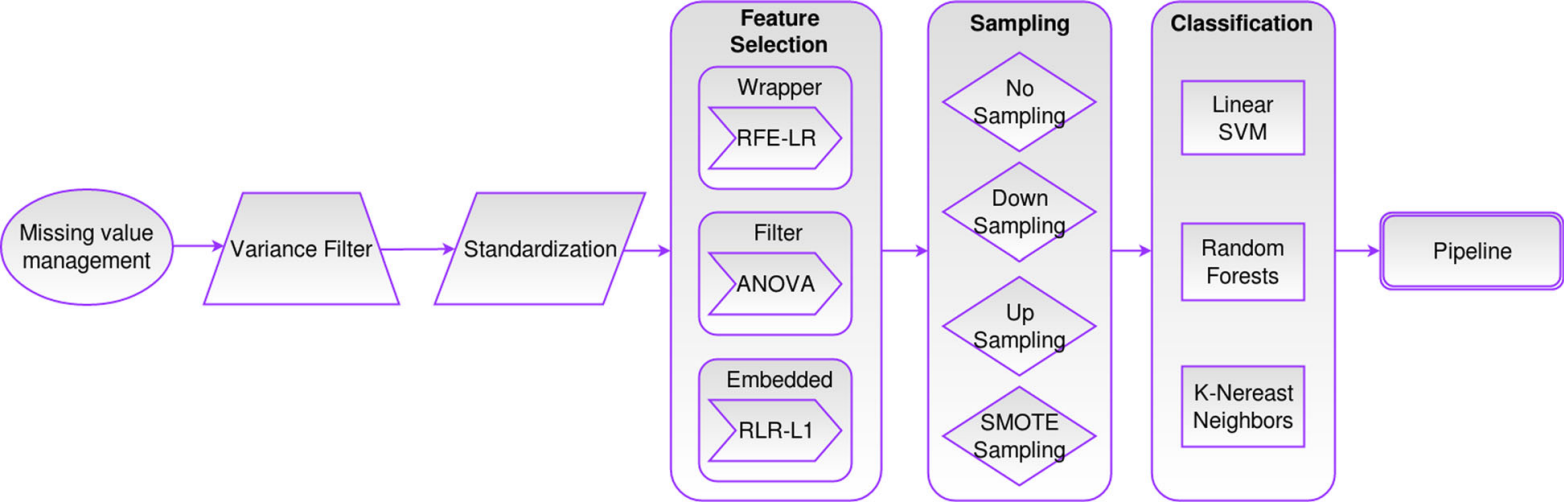
Extended Pipeline Configuration

Figure 1 shows a representation of our “Pipeline Configuration”. The first step consists of a missing value management step. In the presence of missing values in the data-set, imputation is necessary, consisting of replacing any missing value with the mean of the column where the missing value is present. This particular data-set, of treatment response to LC patients, had very few missing values (i.e, Smoker (*n*=1) and ECOG (*n*=3)), treated beforehand using a fast imputation method from the *mice* R library [27]. But having this step in the pipeline makes it easy to be applied to other data-sets with much larger amounts of missing values. Then a variance filter step was added to the pipeline after the imputation step. This is a very simple filter that removes all low-variance features, keeping all features with non-zero variance.

Since we are dealing with data coming from heterogeneous format (SNP data plus clinical and socio-demographic variables), normalization was a crucial step to make measurements comparable. We standardize all the features by removing the mean and scaling to unit variance [28]. This type of data transformation removes statistical errors in repeated measured data. Data are scaled to fall within a small, specified range, thus allowing a fair comparison between different data samples [29].

Considering that we are dealing with high dimensional data, we add a feature selection step to find irrelevant (noisy) or redundant features that do not contribute to the increase of the accuracy/performance of the classification model. We discard these features and keep the relevant ones to move forward in the pipeline process. Feature selection methods are usually classified into three categories: filter, wrapper and embedded methods. Each category of methods has different advantages and disadvantages (see Table 2). We selected one method of each type of feature selection to instantiate the first step of the pipeline: ANOVA as a filter method, recursive feature elimination with logistic regression (RFE-LR) as a wrapper method and regularized L1 logistic regression (RLR-L1) as an embedded method. We selected these specific methods because they are the most popular one’s for each category, and they have been applied to similar data in the context of bioinformatics [9, 30–39].

**Table 2:** Advantages and disadvantages of types of feature selection methods used in the pipeline configuration

FS Methods		
	Advantages	Disadvantages
Filter	They are easily scalable to very high-dimensional data sets.	They do not interact with the classification algorithm.
	They are computationally fast and simple.	Most of this methods are univariate, this is, they consider features independently or only with regard to the target feature, thereby ignoring feature dependencies.
	They are independent of the classification algorithm used in the further model construction.	
Wrapper	They include the interaction between feature subset search and the classification algorithm that is “wrapped”.	They have a higher risk of overfitting, depending on how exhaustive is the feature subset search.
	They take into account feature dependencies.	They are very computationally intensive, especially if the “wrapped” classifier has a high computational cost.
Embedded	They include the interaction between feature subset search and the final classification model constructed.	They depend on the specific learning method of the final model constructed.
	They take into account feature dependencies.	
	They are computationally faster than wrapper methods.	

To deal with the class imbalance distribution present in this type of large-scale data-sets [33, 40], we use one of the pre-processing strategies that Branco et al. proposed in their taxonomy of modelling approaches. We specifically use three types of re-sampling: random under/over-sampling and synthesizing new data using SMOTE-sampling. We also tried as a possibility, keeping the data as it came from the previous pipeline step by not performing any sampling [41].

The final step of the pipeline configuration consists of a ML supervised classification method that builds a model that makes predictions (classification into a given set of categories), based on past observations or labeled training instances. There are several ML classification algorithms in the literature [42]. They use different learning strategies to discriminate samples of different classes. In this study we applied algorithms that fall into three main categories: linear; SVM, tree (non-linear); RF, and distance based methods; KNN [30]. We chose this specific classification methods based on their advantages and disadvantages described in Table 3, and because they are one of the most popular algorithms applied to this type of problems according to several studies. The SVM has been highly used on microarray expression data [43–46] rather than in SNP data. Some few examples of applications use the non-linear radial basis function kernel SVM to analyze the importance of gene-gene interactions on type 2 diabetes (T2D) risk [47] and prostate cancer [48] and to predict hypertension [49], breast cancer susceptibility [50] and chronic fatigue syndrome [51]. As for RF, this algorithm has shown considerable promise using both low and high-dimensional data (from < 100 to > 650K SNPs) identifying associations [52, 53] and disease risk of ischemic heart disease and myocardial infarction [54], as well as classification of T2D [55] or rheumatoid arthritis [56]. Finally the KNN classification method is not very popular in the bioinformatics area, but still it has been used on microarray [57] and gene expression [32, 58] data. It has been also applied to detect selenium resistance of cancer patients [30] and breast cancer classification [59].

**Table 3:** Advantages and disadvantages of classification methods chosen for the pipeline configuration

Classification methods		
	Advantages	Disadvantages
Linear SVM	By introducing the kernel, SVMs gain flexibility in the choice of the form of the threshold separating samples from different classes, which needs not be linear and even needs not have the same functional form for all data, since its function is non-parametric and operates locally.	The lack of transparency of the results.
	Since the kernel implicitly contains a non-linear transformation, no assumptions about the functional form of the transformation, which makes data linearly separable, is necessary.	The SVM moves the problem of over-fitting from optimizing the parameters to model selection.
	SVMs provide a good out-of-sample generalization, if the parameters (C for example) are appropriately chosen. This means that, by choosing an appropriate generalization grade, SVMs can be robust, even when the training sample has some bias.	
	SVMs deliver a unique solution, since the optimality problem is convex.	
RF	It decides the final classification by voting, decreasing the variance of the model without increasing the bias.	It is hard to visualize the model or understand why it predicted something, as compared to a single decision tree.
	It uses a random subset of features at each node of the decision trees, to identify the best split among this subset, and the subsets are different in each node. This is to avoid the most powerful features being selected too frequently in each tree, making them more correlated to each other.	A large number of trees may make the algorithm slow for real-time prediction.
	It is fast even on large data-sets.	RFs have been observed to over-fit for some data-sets with noisy classification/regression tasks.
	It gives estimates of what variables are important in the classification.	
KNN	The cost of the learning process is zero.	The algorithm must compute the distance and sort all the training data at each prediction, which can be slow if there are a large number of training examples.
	No assumptions about the characteristics of the concepts to learn have to be done.	The algorithm does not learn anything from the training data, which can result in the algorithm not generalizing well and also not being robust to noisy data.
	Complex concepts can be learned by local approximation using simple procedures.	Changing *k* can change the resulting predicted class label.

The purpose of a machine learning pipeline is to assemble several ML steps into one. This is useful as they can be cross-validated together while setting different parameters. Thus, pipelines help to avoid leaking statistics from test data into the trained model in cross-validation, by ensuring that the same samples are used to train the pipeline steps and that training and test data go through identical feature processing steps. Pipelines are available in main programming language tools for machine learning [28, 60, 61] and they have already been used in previous research articles [62, 63] such as for discriminant pathway identification or quantitative phenotype prediction.

### ML framework design

This framework splits the data in chromosomes, and applies the pipeline configuration to each chromosome separately as an initial partial analysis. We use the stability score calculated for each feature as a “filter” to select the most important and “stable” features from each chromosome. Using the latter “filtered” features, “filtered/merged” training and test data-sets are created and used to construct a unique “final model”. This model can now take advantage of features from the whole genome. Our proposed framework follows the idea of model selection using *k*-fold cross-validation (CV) in both, the partial analysis done with each chromosome and the final analysis done with the “filtered/merged” data.

Using all possible combinations of instantiations from each step of the pipeline configuration, 36 different experiments were executed. Three feature selection methods: ANOVA, RFE-LR, RLR-L1, by four sampling techniques: No sampling, Down-sampling, Up-sampling and SMOTE-sampling, by three classification algorithms: Linear SVM, RF, KNN.

First the whole original data-set (containing features from the 22 chromosomes) was split into a test and a “preliminary” data-set that was split again into training and stability data-sets.

The partial analysis that was done with the data of each of the 22 chromosomes separately, is described as follows. For a certain pipeline instantiation, a *k*-fold CV with hyper-parameter tuning is executed using the training data-set of the chromosome under analysis. From this process we obtain what we call the “partial model”. We use this “partial model” to calculate the stability score for each feature which is initialized with a value of zero.

*S* samples/shuffles without replacement of *T* percent of the stability data-set are generated. For each sample/shuffle the “partial model” is re-fitted. For each feature, if the feature was selected by the feature selection step of the “partial model”, the stability score is increased by one unit. At the end of this iterative process each feature will have a stability score ranging between zero and *S*. The larger the score, the more stable the feature will be considered.

Finally, the features from the chromosome under analysis are filtered and only the one’s with a stability score greater or equal to a user-defined threshold *W* are kept to create new “filtered/merged” versions of the training and test data-sets with variants from all the genome.

Using the “filtered/merged” training data-set we perform again *k*-fold CV with hyper-parameter tuning to create the “final model”, which is evaluated using the “filtered/merged” test data-set.

**Fig. 2:**
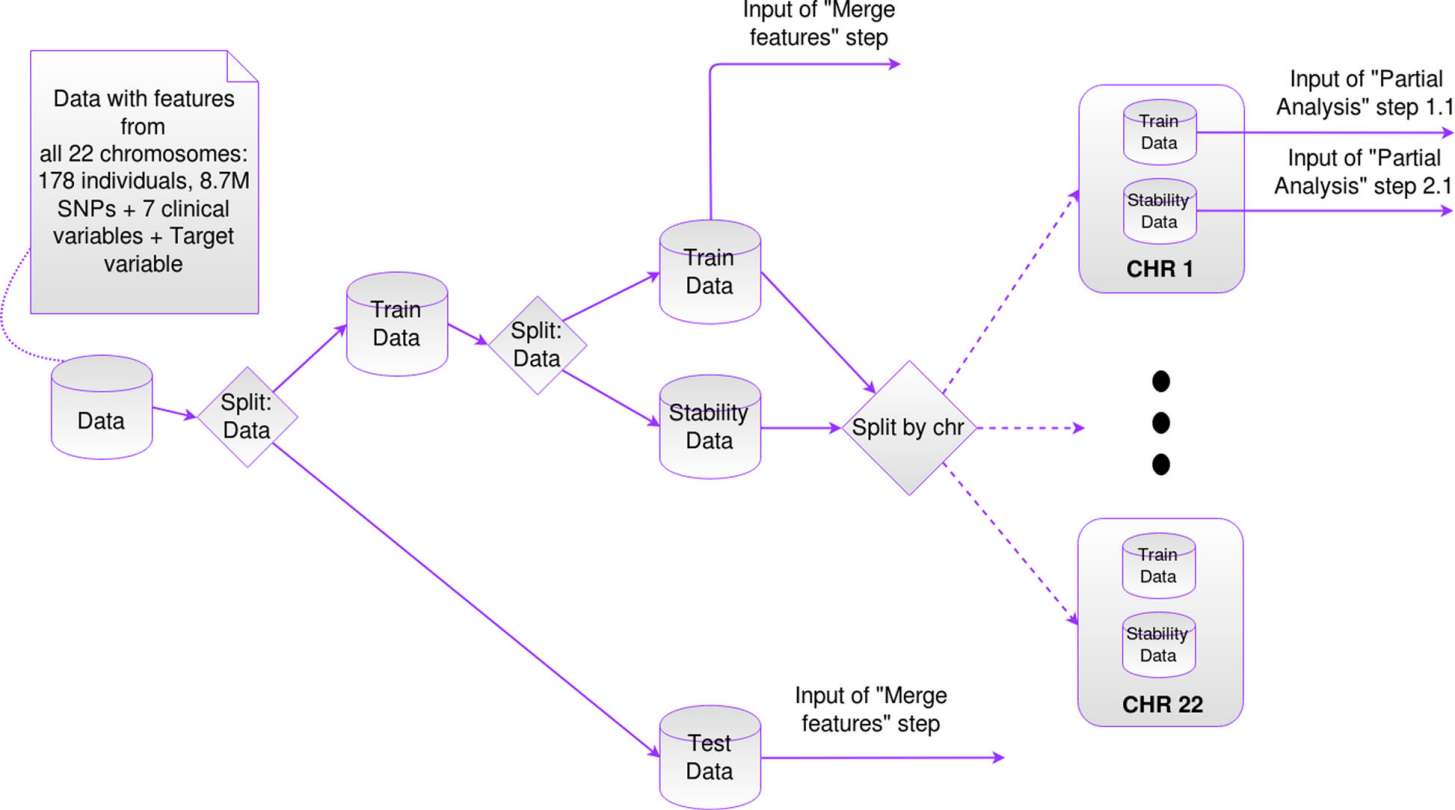
Initial steps of “General Framework”

**Fig. 3:**
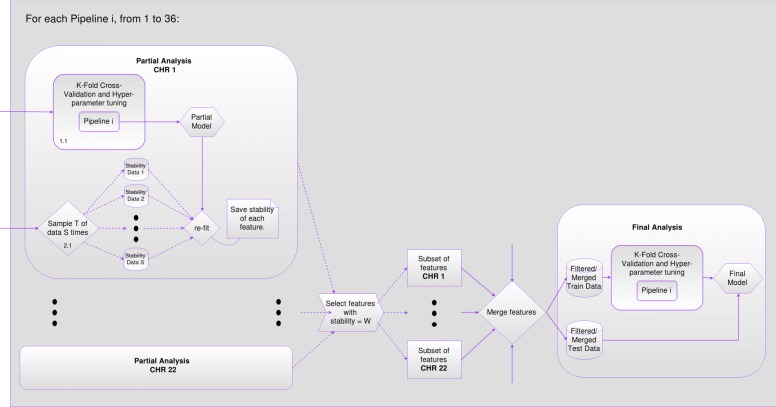
Main loop of “General Framework” where the “Partial Analysis” is executed for each chromosome in the genome and results are finally merged in the “Final Analysis”

**Fig. 4:**
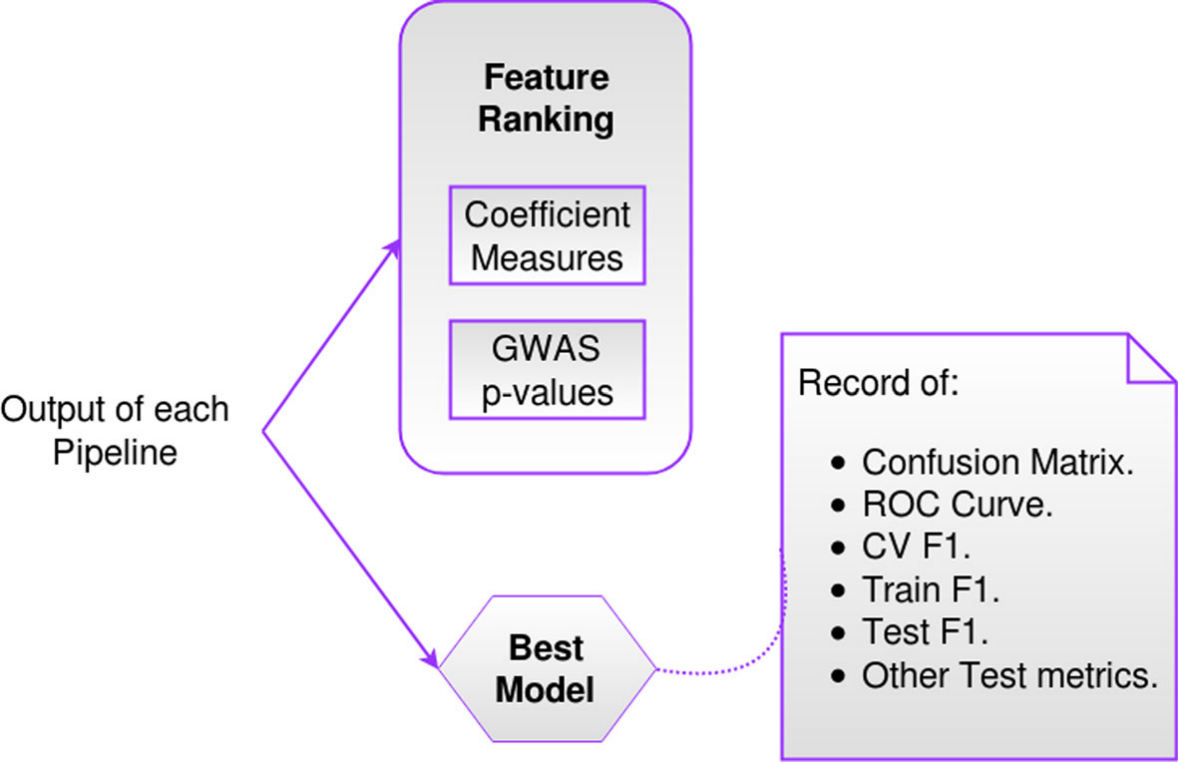
Output of “General Framework” corresponding to each of the 36 pipeline configurations

We are aware that filtering the features of each chromosome using the stability score (to create a “filtered/merged” training and test data-sets), outside the final CV loop, introduces bias to the process of model selection, because part of the data has been seen before during model selection of each chromosome model. To reduce this bias, we propose the use of an independent stability data-set. This stability score filter was introduced mainly to be able to create a “final model” that uses features from all chromosomes (the most stable ones), and be able to take into account possible interactions and correlations between SNPs of different chromosomes. We finally test the predictive power of the “final model” with the separate and independent “filtered/merged” test set that has not been used during model selection in either of the partial o final analysis. Figures 2, 3 and 4 show a graphical version of the general framework.

Using the “final model” we keep track of some metrics to rank over the SNPs, based on characteristics of specific instantiations of the classification step of the pipelines. For example, if the classifier of the pipeline in analysis is a Linear SVM, we save the values of the weights assigned by the algorithm to each feature. In a similar way, for the case of RF, we keep record of the variable importance metric [64] associated to each feature while using this classification model. In the case of KNN, since there is no intrinsic measure associated to the method from which features can be ranked, we use measures associated to the previous feature selection method of the pipeline applied to the data, for example, when using ANOVA filter feature selection, we use the *p*-values calculated from the statistical test; when using the RFE-LR wrapper method, we use the absolute value of the coefficients of the wrapped logistic regression (LR) associated to each feature. Similarly the absolute value of the coefficients of the RLR-L1 embedded method are used. The signs of the coefficients were also stored so that we could measure the effect of the feature in the classification result.

It is important to stand out that the same instantiation of the extended pipeline is used in the partial analysis by chromosome and in the final analysis using the “filtered/merged” training and test data-sets. This is a criterion defined by us and not a limitation. Since both pipelines are validated using *k*-fold CV and grid-search (for hyper-parameter tuning), each pipeline may have a different hyper-parameter settings.

In our knowledge, performing a partial analysis in 22 pieces, for each chromosome, and merging for a final analysis for the whole genome feature analysis is not reported anywhere. Furthermore, including all ML steps (feature selection, sampling and classification) for every CV fold, make our approach for a unique manageable pipeline, to be applicable to complex studies for extract maximum of biological knowledge.

#### Data setup

To perform model selection and evaluation as explained in the ML Framework Design section, the data-set was split into training, stability and test sets as follows. The original data-set was split into 20–80% corresponding to test and a “preliminary” training data-sets with 36 and 142 samples respectively. The “preliminary” training data-set was split again into 50–50% corresponding to the training and stability data-sets with 71 samples each.

All of the different splits were performed in a stratified way to ensure the same proportion of individuals of each class, in training, stability and test sets, as in the original data-set.

#### Parametrization setup

The pipeline was validated using *k* = 5 during the *k*-fold CV along with the F1 weighted measure as scoring function [65]. We use the latter scoring function due to the nature and distribution of the data, since we know beforehand that classes are imbalanced and we want to give equal importance to the precision and recall of both classes. The tuning of hyper-parameters associated to each step of the pipelines was performed using a grid-search. The different parameters tried are shown in Table 4. The value of the *k* of the cross-validation process as well as the different ranges of values used during grid-search, are the standard set of parameters normally used in training these algorithms.

**Table 4:** Parameters tested using grid-search and 5-fold CV. EFD refers to the “Extended Framework Design”

Pipeline step	Parameter options
ANOVA	EFD (Partial analysis): percentile = 2% of total # of variables
	EFD (Final analysis): percentile = 10% of total # of variables
	LR penalty = ’l1’
	C = 1
RFE-LR	RFE EFD (Partial analysis):
	n_features_to_select = 2% of total # of variables,
	step = 4%
	EFD (Final analysis):
	n_features_to_select = 10% of total # of variables,
	step = 10%
RLR-L1	penalty = ’l1’
	EFD (Partial analysis): C = [100, 500, 1000, 1500, 5000, 10000]
	EFD (Final analysis): C = [100, 500, 1000, 1500, 5000, 10000]
	threshold = 1*e*−10
Linear SVM	C = [0.001, 0.01, 0.1, 1, 10, 100, 1000]
RF	n_estimators = [30,47, 75, 119, 189, 299, 475, 753,1194,1892,2999]
KNN	n_neighbors = [5, 20, 35, 50]

The *percentile* parameter in ANOVA corresponds to the percentage of features to keep as a result of the feature selection step. For the RFE-LR, the parameters related to the LR model “wrapped” by the RFE method remained static with a L1 *penalty* (that contributes to reduce the number of features in the LR “wrapped” model) and the default *C* value equal to 1, that refers to to the inverse of regularization strength. As for the RFE parameters, the *n_features_to_select* refers to the percentage of features to keep at the end of the iterative search, and the *step* parameter corresponds to the number of features to drop at each iteration. In the case of RLR-L1, as the name implies, a L1 *penalty* was used, and a range of values were tried for the regularization parameter *C*. The *threshold* parameter refers to the threshold value used for feature selection. Features whose LR coefficient is greater or equal are kept while the others are discarded. For Linear SVM the *C* parameter refers to the penalty parameter of the error term. In both of the latter cases using the *C*, the smaller the values, the stronger the regularization. The *n_estimators* parameter in RF refers to the number of trees in the forest and *n_neighbors* in KNN is the number of neighbors to take into account in the neighbors voting step of the classifier.

*S* = 100 different samplings/shuffles without replacement of *T* = 80% of the stability data-set were used to record the stability score of all the features of each chromosome. Instantiating *W* = 100, features from each chromosome were filtered and merged together to create a “filtered/merged” training and test data-sets containing features from the whole genome. Setting *W* = 100 is restrictive, but it is on purpose because we are aiming to keep the most stable features from all of the 22 chromosomes.

### Intersection analysis with GWAS catalog

Once the most relevant SNPs are identified from the 36 experiments of the pipeline, we compare these SNPs with the associated SNPs that have been reported in the literature in LC studies. For this purpose, we consider the SNPs identified from the subset of the “final models” with *CV F1* scores between the highest score and the latter minus 0.1. This subset correspond to the top 20 pipelines ranked by *CV F1* score.

We later contrasted/intersected these lists with the list of SNPs selected by the last step of the top 20 pipelines, i. e. the classifiers, to create three new lists: “ML Rank cat ALL”, “ML Rank cat LUNG” and “ML Rank cat CANCER”.

We downloaded the v1.0 (release date: 2017-07-31) with all associations of the GWAS Catalog of the National Human Genome Research Institute (NHGRI) website [66]. From the original 44,738 entries, we discard entries representing SNP interactions, and keep only 32,990 entries corresponding to unique chromosomal positions and terms. We will call the latter list the “GWAS cat ALL” list. From this list, we filtered reported terms to define a list with a narrow definition, “GWAS cat LUNG” (i.e. Pulmonary, Lung, NSCLC, Response, Chemotherapy, Platinum Survival) (Table 5) and other with a extended analysis, “GWAS cat CANCER” (i.e. Pulmonary, Lung, NSCLC, SCLC, Cancer, Response, Chemotherapy, Platinum, Survival) (Tables 6 and 7). All included associations were with a *p*-value under 10*e*−6 threshold.

**Table 5:** LC related traits from the GWAS Catalog v1.0 (release date: 2017-07-31)

LC related traits
Pulmonary function
Lung adenocarcinoma
Lung cancer
Lung cancer (DNA repair capacity)
Lung cancer (smoking interaction)
Non-small cell lung cancer
Non-small cell lung cancer (recurrence rate)
Non-small cell lung cancer (survival)
Response to platinum-based agents
Response to platinum-based chemotherapy (carboplatin)
Response to platinum-based chemotherapy (cisplatin)
Response to platinum-based chemotherapy in non-small-cell lung cancer
Adverse response to chemotherapy (neutropenia/leucopenia) (cisplatin)

**Table 6:** 1/2 Cancer related traits from the GWAS Catalog v1.0 (release date: 2017-07-31)

Cancer related traits
Adverse response to chemotherapy (neutropenia/leucopenia) (cisplatin)
Adverse response to chemotherapy in breast cancer (alopecia)
Adverse response to chemotherapy in breast cancer (alopecia) (anti-microtubule)
Adverse response to chemotherapy in breast cancer (alopecia) (cyclophosphamide+doxorubicin+/-5FU)
Adverse response to chemotherapy in breast cancer (alopecia) (cyclophosphamide+epirubicin+/-5FU)
Adverse response to chemotherapy in breast cancer (alopecia) (docetaxel)
Adverse response to chemotherapy in breast cancer (alopecia) (paclitaxel)
Anthracycline-induced cardiotoxicity in childhood cancer
Bladder cancer
Bladder cancer (smoking interaction)
Body mass index (change over time) in cancer
Body mass index (change over time) in cancer or chronic obstructive pulmonary disease
Body mass index (change over time) in gastrointestinal cancer
Body mass index (change over time) in gastrointestinal cancer or chronic obstructive pulmonary disease
Body mass index (change over time) in lung cancer
Body mass index (change over time) in lung cancer or chronic obstructive pulmonary disease
Breast cancer
Breast cancer (early onset)
Breast cancer (estrogen-receptor negative
Breast cancer (estrogen-receptor negative)
Breast cancer (estrogen-receptor positive)
Breast cancer (male)
Breast cancer (menopausal hormone therapy interaction)
Breast cancer (prognosis)
Breast cancer (survival)
Breast Cancer in BRCA1 mutation carriers
Breast cancer in BRCA2 mutation carriers
Breast cancer-free interval (treatment with aromatase inhibitor)
Cancer
Cancer (pleiotropy)
Cardia gastric cancer
Cervical cancer
Colon cancer
Colorectal cancer
Colorectal cancer (alcohol consumption interaction)
Colorectal cancer (aspirin and/or NSAID use interaction)
Colorectal cancer (calcium intake interaction)
Colorectal cancer (diet interaction)
Colorectal cancer (interaction)
Colorectal cancer (oestrogen-progestogen hormone therapy interaction)
Colorectal or endometrial cancer
Disease-free survival in breast cancer
Docetaxel-induced peripheral neuropathy in metastatic castrate-resistant prostate cancer
Endometrial cancer
Epithelial ovarian cancer
Erectile dysfunction and prostate cancer treatment
Esophageal cancer
Esophageal cancer (alcohol interaction)
Esophageal cancer (squamous cell)
Esophageal cancer and gastric cancer
Esophageal squamous cell cancer (length of survival)
Estradiol plasma levels (breast cancer)
Estrogen receptor status in breast cancer
Estrogen receptor status in HER2 negative breast cancer
Estrone conjugates/estrone ratio in resected early stage estrogen-receptor positive breast cancer
Estrone/androstenedione ratio in resected early stage-receptor positive breast cancer
Gallbladder cancer
Gastric cancer
Lobular breast cancer (menopausal hormone therapy interaction)
Lung adenocarcinoma
Lung cancer
Lung cancer (asbestos exposure interaction)
Lung cancer (DNA repair capacity)
Lung cancer (smoking interaction)
Multiple cancers (lung cancer
Multiple keratinocyte cancers
Non-cardia gastric cancer

**Table 7:** 2/2 Cancer related traits from the GWAS Catalog v1.0 (release date: 2017-07-31)

Cancer related traits
Non-melanoma skin cancer
Non-small cell lung cancer
Non-small cell lung cancer (recurrence rate)
Non-small cell lung cancer (survival)
Obesity in adult survivors of childhood cancer exposed to cranial radiation
Obesity in adult survivors of childhood cancer not exposed to cranial radiation
Oral cavity and pharyngeal cancer
Oral cavity cancer
Oropharynx cancer
Ovarian cancer
Ovarian cancer in BRCA1 mutation carriers
Pancreatic cancer
Plasma androstenedione levels in resected early stage-receptor positive breast cancer
Plasma estrone conjugates levels in resected early stage estrogen-receptor positive breast cancer
Plasma estrone levels in resected estrogen-receptor positive breast cancer
Platinum-induced myelosuppression in non-small cell lung cancer
Progression free survival in metastatic colorectal cancer (CAPOX-B vs CAPOX-B plus cetuximab)
Progression free survival in metastatic colorectal cancer (treatment interaction)
Prostate cancer
Prostate cancer (early onset)
Prostate cancer (interaction)
Prostate cancer (survival)
Prostate cancer aggressiveness
Pulmonary function
Response to carboplatin and paclitaxel in ovarian cancer (Caspase 3/7 EC50)
Response to carboplatin and paclitaxel in ovarian cancer (MTT IC50)
Response to carboplatin in ovarian cancer (MTT IC50)
Response to chemotherapy in breast cancer (hypertension) (bevacizumab)
Response to chemotherapy in breast cancer hypertensive cases (cumulative dose) (bevacizumab)
Response to gemcitabine in pancreatic cancer
Response to irinotecan and platinum-based chemotherapy in non-small-cell lung cancer
Response to irinotecan in non-small-cell lung cancer
Response to paclitaxel in ovarian cancer (Caspase 3/7 EC50)
Response to paclitaxel in ovarian cancer (MTT IC50)
Response to Pazopanib in cancer (hepatotoxicity)
Response to platinum-based agents
Response to platinum-based chemotherapy (carboplatin)
Response to platinum-based chemotherapy (cisplatin)
Response to platinum-based chemotherapy in non-small-cell lung cancer
Response to platinum-based neoadjuvant chemotherapy in cervical cancer
Response to radiotherapy in cancer (late toxicity)
Response to radiotherapy in prostate cancer (overall toxicity)
Response to radiotherapy in prostate cancer (toxicity
Response to radiotherapy in prostate cancer (toxicity
Response to radiotherapy in prostate cancer (toxicity
Response to radiotherapy in prostate cancer (toxicity)
Response to tamoxifen in breast cancer
Small-cell lung cancer (survival)
Survival in colon cancer
Survival in colorectal cancer
Survival in colorectal cancer (distant metastatic)
Survival in colorectal cancer (non-distant metastatic)
Survival in endocrine treated breast cancer (estrogen-receptor positive)
Survival in head and neck cancer
Survival in microsatellite instability low/stable colorectal cancer
Survival in rectal cancer
Testicular cancer
Testicular germ cell cancer
Thyroid cancer
Thyroid cancer (Papillary
Urinary bladder cancer
Urinary symptoms in response to radiotherapy in prostate cancer

### Functional SNP analysis

The key features identified by the 20 top models were explored with the Regulomedb [67] and GTEx databases [68] by using the haploR package [69]. The Regulomedb database offers a score from 1 to 7 for each variant, the lower the score, the more likely the variant has a functional activity. The GTEx databases provide information of the relationship between the expression levels of genes and genetic variation from previous studies involving human tissues from donors. This relationship is known by the expression quantitative trait loci (eQTL). We focus the analysis on the eQTL data from the lung tissues. The GTEx portal shows *p*-values from the eQTL analysis and also “m-values” derived from the meta-analysis of multiple tissues performed by METASOFT [70]. The larger the m-value, the more likely the effect exists in each study.

#### Infrastructure

All the calculations were performed in a computer with the following characteristics: 48 GB of RAM and 32 GB of Swap Memory, 12 Intel®;Cores™i7-5820K CPU @ 3.30GHz, under Ubuntu 16.04.2 LTS Linux distribution. The general framework and pipeline were implemented using Python 3.5.2, and Scikit-learn 0.19. Scikit-learn is a Python module that integrates a wide range of state of the art ML algorithms for medium-scale supervised and unsupervised problems [28]. Even though everything was executed in a single node/computer, we took advantage of Scikit-learn’s parallel implementations (in almost all of the algorithms and techniques used), to reach the maximum potential of the architecture described above. In execution time, all the 36 pipeline experiments lasted in total, approximately three and a half weeks. Specific times for each experiment can be seen in detail in Additional file 1. Regarding the precision in the implementation of our algorithms, it is 10*e*−12, which is well below the numeric tolerance and parameters used in our training algorithms. The final results obtained are therefore not affected by this numeric tolerance.

## Results

### ML framework

A total of 36 experiments were executed following the ML framework showed in Figs. 2, 3 and 4. Each application of the pipeline was validated using *k*-fold CV, along with F1 weighted measure as scoring function. Grid-search was combined during *k*-fold CV to find the best hyper-parameter setting for a specific pipeline using a training set, and afterwards having chosen a specific setting (the one with highest *CV F1* score, the “final model”), we test the predictive power of the model with a separate and independent test set (for which sampling has not been applied, preserving the original distribution of the data) of 36 samples. Using the confusion matrix, we record several metrics such as CV F1, Train F1, and Test F1, Accuracy, Precision and Recall. We also recorded metrics associated specifically to each class and the model parameters used for each pipeline.

**Fig. 5:**
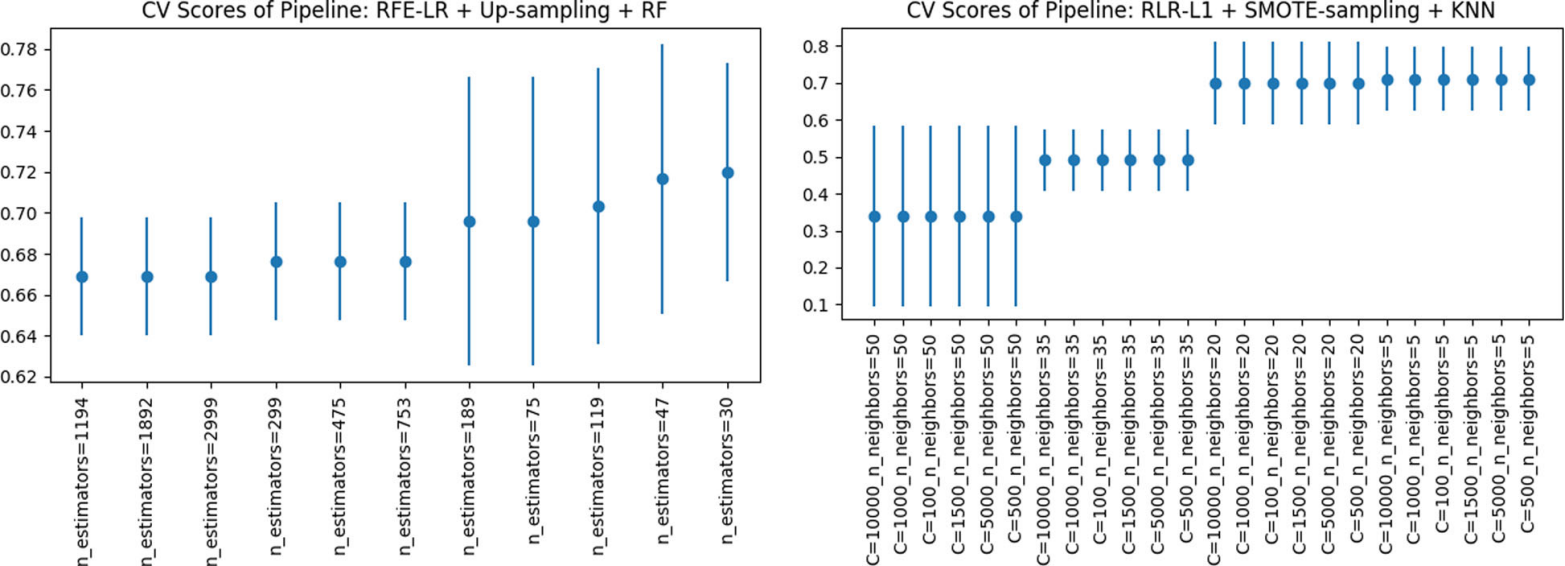
Parameter sensitivity analysis of top 2 pipeline configurations with the highest *CV F1* score obtained during model selection

**Fig. 6:**
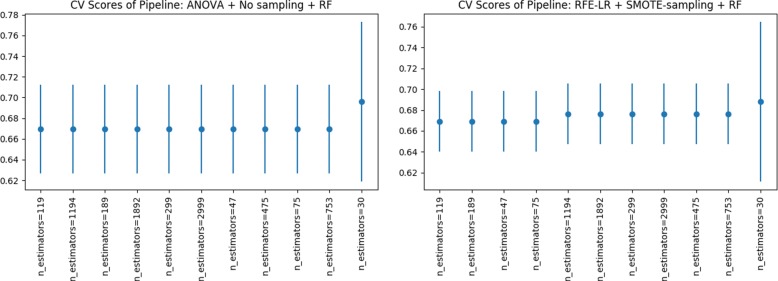
Parameter sensitivity analysis of pipeline configurations in third and fourth positions with the highest *CV F1* score obtained during model selection

**Fig. 7:**
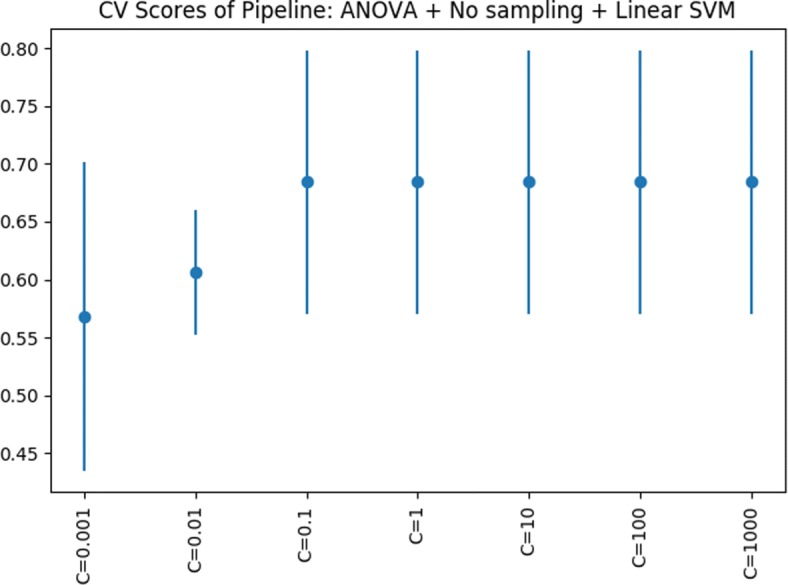
Parameter sensitivity analysis of pipeline configuration in fifth position with the highest *CV F1* score obtained during model selection

Figures 5, 6 and 7 show the *CV F1* scores for different parameter settings for the top five pipelines. Figure 5 (right) shows an interesting parameter sensitivity trend were we can see that the alteration of the regularization parameter of the LR model, does not have much effect on the performance scores obtained, irrespective from the “n_neighbors” parameter of the KNN classifier. On the other hand, we see a considerable difference in *CV F1* scores when varying the KNN’s “n_neighbors” parameter. Regarding the models with RF as classification step (Fig. 5 (left) and Fig. 6), we consistently see that the smallest the number of trees, the better performance scores. Finally, Fig. 7 shows a positive relationship; the larger the SVM’s regularization parameter, the larger the *CV F1* score; up to “C =0.1”, where an increment of the “C” parameter do not increase the *CV F1* score and remains constant. Guided by the results shown in the latter plots, in future improvements grid-search analysis, we recommend to use “n_estimators < 200” for RF, “n_neighbors <=20” for KNN’s and “C > 1” for SMV’s.

Table 8 shows the top 5 pipeline configurations with the highest CV F1 score obtained during model selection. The scores from the rest of the experiments and a detailed description of the meaning of the used evaluation metrics can be found in the Additional files 1 and 2. Focusing on the 36 experiments, it can be seen that more than half of the pipeline instantiations have CV F1 scores above the mean (mean = 0.593), with decent values from the practical point of view, considering the complexity of the classification problem, the high number of features we are dealing with and the small amount of available training data.

**Table 8:** Model selection and evaluation metrics (general and per class) of top 5 models from 36 possible instantiations of pipeline using LC data-set

	FS	Sampling	Classifier	CV F1	CV Precision	CV Recall	Train	Test	Test	Test	Test	Test	Test	Test	Test	Test	Model
				Mean ± Std	Mean ± Std	Mean ± Std	F1	F1	Precision	Recall	F1 (0)	Precision (0)	Recall (0)	F1 (1)	Precision (1)	Recall (1)	Parameters
1	RFE-LR	Up-sampling	RF	0,72 ± 0,054	0,686 ± 0,102	0,79 ± 0,039	1	0,722	0,778	0,729	0,871	0,964	0,794	0,2	0,125	0,5	n_estimators =30
2	RLR-L1	SMOTE-sampling	KNN	0,712 ± 0,087	0,68 ± 0,122	0,762 ± 0,066	0,777	0,741	0,806	0,844	0,889	1	0,8	0,222	0,125	1	n_neighbors =5,
																	C =100
3	ANOVA	No sampling	RF	0,698 ± 0,077	0,651 ± 0,12	0,776 ± 0,061	1	0,652	0,722	0,595	0,839	0,929	0,765	0	0	0	n_estimators =30
4	RFE-LR	SMOTE-sampling	RF	0,689 ± 0,077	0,648 ± 0,119	0,761 ± 0,071	1	0,681	0,778	0,605	0,875	1	0,778	0	0	0	n_estimators =30
5	ANOVA	No sampling	Linear SVM	0,687 ± 0,113	0,687 ± 0,136	0,707 ± 0,112	1	0,811	0,833	0,823	0,9	0,964	0,844	0,5	0,375	0,75	C =0.1

They are ordered by CV F1. FS stands for feature selection, Cv for cross-validation, F1 is the measure of model evaluation defined as: Precision x Recall / (Precision + Recall). Precision is the proportion of examples classified as positive that are truly positive and Recall the proportion of truly positive examples that are classified as positive. Std stands for standard deviation. Train indicates we used the training set to compute the evaluation metric and Test if we used the test set. (0) indicates it’s an evaluation metric for class 0 and (1) for class 1

**Fig. 8:**
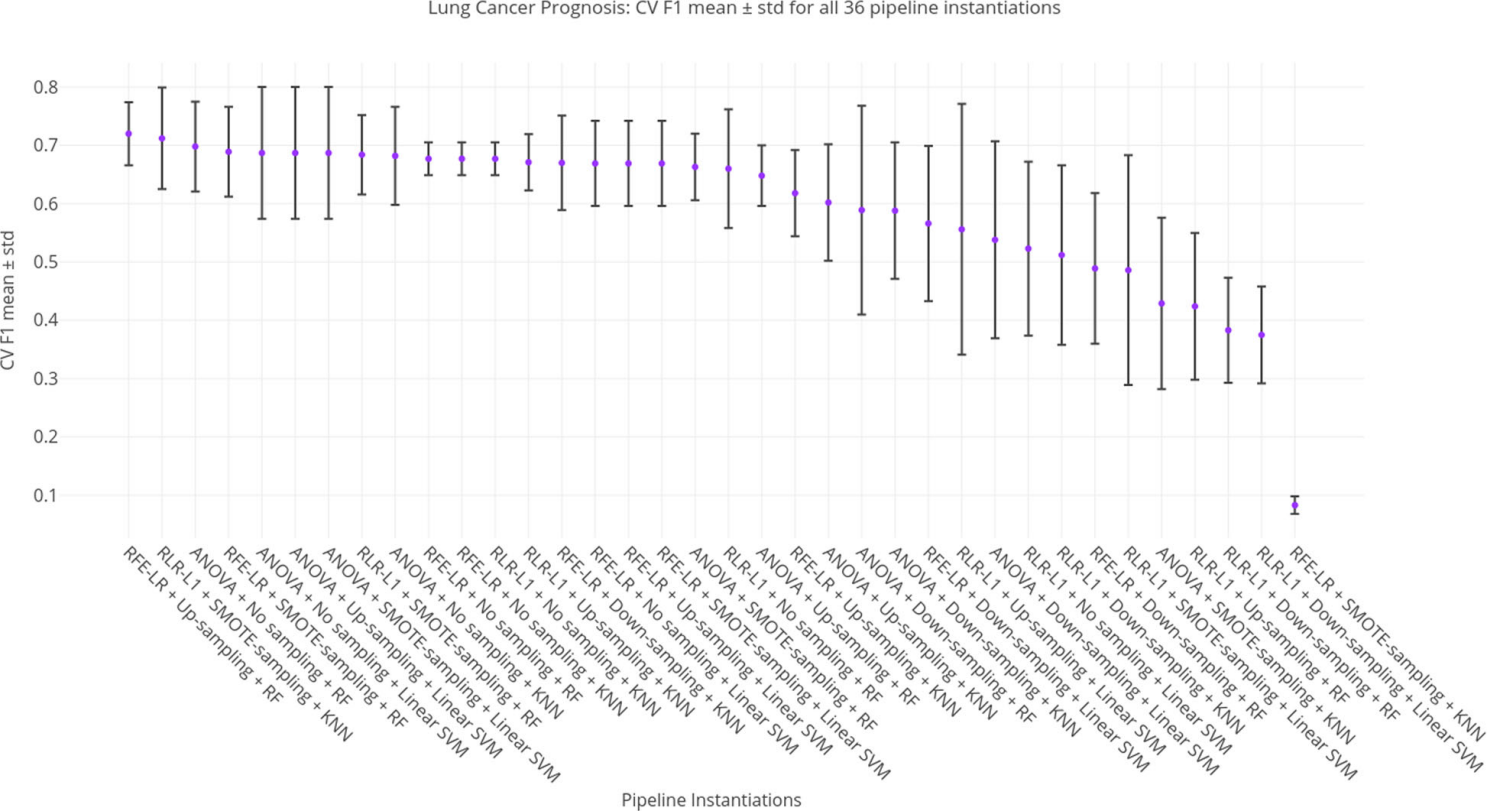
CV F1 mean scores with their corresponding standard deviations for all 36 pipeline instantiations using LC data-set

Regarding the standard deviations (sd) from the *CV F1* scores, 58% of the models have a sd below 0.1. It shows that the model selection process (CV) is robust and we are confident that these values are close to the real scores. This is also a sign that the models are stable and trustworthy. Figure 8 shows an error bar plot for each model, where the purple dots represent the mean *CV F1* score and the black bars the standard deviation of the 5-fold CV process of the best setting found during grid-search.

Detailing the *CV Precision* score, it can be seen that these metrics tend to have smaller mean values and larger sd than the *CV F1* and *CV Recall* scores. Whereas, the *CV Recall* scores have larger mean values and smaller sd than the *CV F1* and *CV Precision* scores. This last phenomenon is interesting because since we are dealing with an imbalanced class problem, the *Recall* is a very important metric to take into account. From a medical and/or biological point of view, having high values of false negatives (*FN*) is bad. In this particular analysis, we want to avoid predicting that a certain patient responds to treatment, when in reality he/she does not, because it would imply making false conclusions about survival chances if incorrect treatment is chosen. On the other hand, having too many false positives (*FP*) is not as severe as the latter case. In these cases, what usually happens is that further medical tests are done to corroborate the result before providing any treatment of choice.

Almost all the *Test F1* scores are very close to their corresponding *CV F1* scores. However, in some cases, the *Test F1* score is larger than the *CV F1* score, but this is due to the particular sampling of the folds during CV.

From the top five pipeline models, RF seems to outperform the other classification methods, regardless of the feature selection and sampling methods it was paired with, but this does not seem to be a general conclusion when we detail the whole table of 36 results (see Additional file 1).

**Fig. 9:**
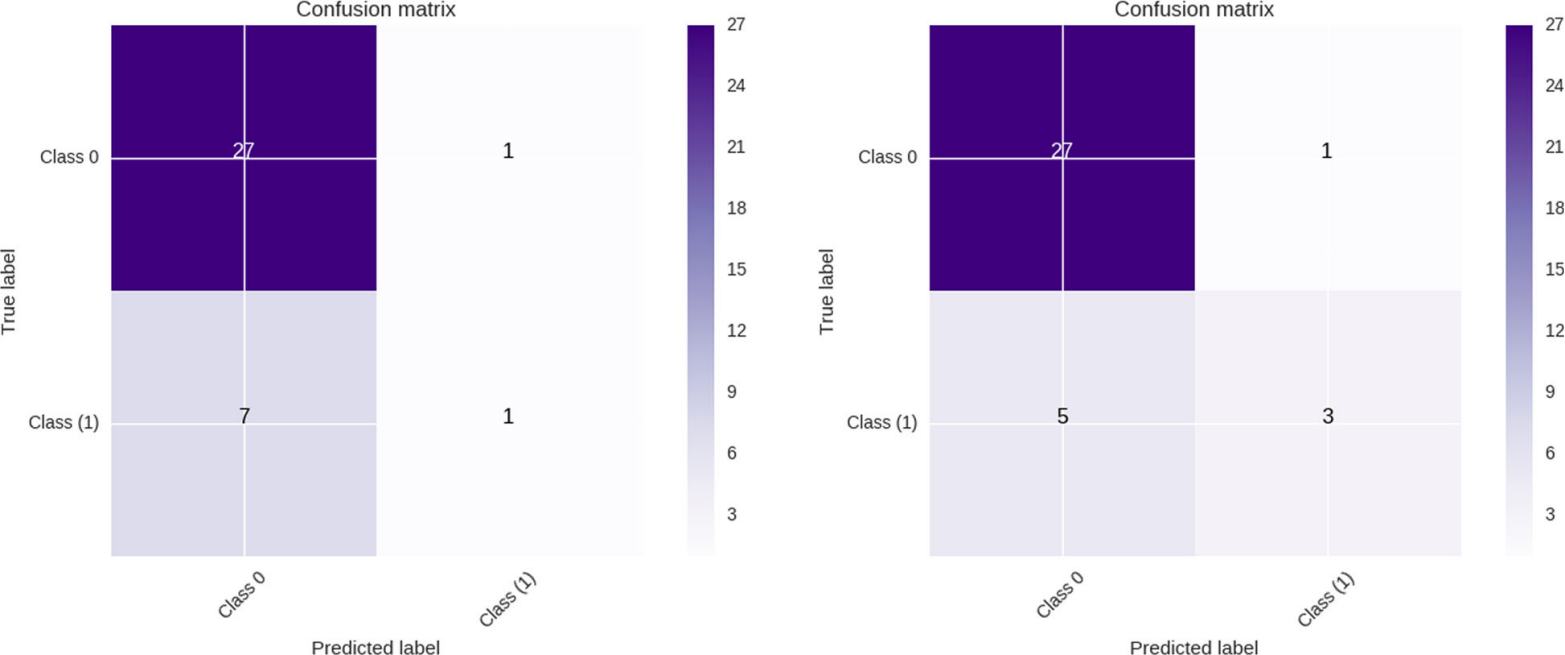
Confusion matrix of LC test data-set using first pipeline model: RFE-L1 + Up-sampling + RF (left) and fifth pipeline model: ANOVA + No sampling + Linear SVM (right)

The pipeline configuration with the highest *CV F1* score consists of applying recursive feature elimination with logistic regression as the feature selection step, followed by up-sampling and finally using random forest as a non-linear classification algorithm (RFE-LR + Up-Sampling + RF). We compare the results obtained by the latter model with the one’s corresponding to the fifth model: ANOVA + No sampling + Linear SVM, since this model shows to have higher values in the *Test* scores. Figure 9 shows the confusion matrices of the first and fifth model. Both pipeline models are able to classify accurately almost all of the test samples from the negative (Class 0) test samples. The first model struggles severely with the positive class, being able to predict correctly only one of the test samples. The fifth pipeline model performs better, being able to correctly classify almost half of the positive (Class 1) test samples.

### Intersection analysis with GWAS catalog

The pipeline model with the highest *CV F1* had a score of 0.72. We performed the GWAS intersection analysis with models included in the interval [0.62, 0.72] corresponding to the top *CV F1* score minus 0.10. This criteria includes the top 20 pipeline models (*CV F1* score >= 0.65). All of them have *CV F1* score larger the mean of the same score of all the experiments (0.59). These models identify 1,224 unique SNPs.

Table 9 shows for each pipeline the number of SNPs intersected with the “GWAS cat ALL”, “GWAS cat LUNG” and “GWAS cat CANCER” lists.

**Table 9:** Results of analysis of intersection of relevant SNPs given by the ML models, with GWAS Catalog records associated with LC and Cancer

Pipeline	# of	ML Rank	ML Rank	ML Rank
	features	cat ALL	cat LUNG	cat CANCER
RFE-LR + Up-sampling + RF	257	0	0	0
RLR-L1 + SMOTE-sampling + KNN	13	0	0	0
ANOVA + No sampling + RF	144	0	0	0
RFE-LR + SMOTE-sampling + RF	238	1	0	0
ANOVA + No sampling + Linear SVM	193	0	0	0
ANOVA + Up-sampling + Linear SVM	193	0	0	0
ANOVA + SMOTE-sampling + Linear SVM	193	0	0	0
RLR-L1 + SMOTE-sampling + RF	3	0	0	0
ANOVA + No sampling + KNN	95^a^	0	0	0
RFE-LR + No sampling + RF	305	0	0	0
RFE-LR + No sampling + KNN	148^b^	2	0	0
RLR-L1 + No sampling + KNN	17	0	0	0
RLR-L1 + Up-sampling + KNN	16	0	0	0
RFE-LR + Down-sampling + KNN	148^b^	2	0	0
RFE-LR + No sampling + Linear SVM	148^b^	2	0	0
RFE-LR + Up-sampling + Linear SVM	148^b^	2	0	0
RFE-LR + SMOTE-sampling + Linear SVM	148^b^	2	0	0
ANOVA + SMOTE-sampling + RF	193	0	0	0
RLR-L1 + No sampling + RF	17	0	0	0
ANOVA + Up-sampling + RF	193	0	0	0

^a^corresponds to 5% of the top features selected by the ANOVA feature selection method. ^b^corresponds to 0,1% of the top features selected by the RFE-LR feature selection method

All intersections with both lists “GWAS cat LUNG” and “GWAS cat CANCER”, for the top 20 pipeline models were empty. Only for a couple of cases, the intersection with the “GWAS cat ALL” list gave non-empty results.

These results suggest that none of the SNPs identified as relevant by the combination of ML methods applied in this study, using the top 20 pipeline models, were previously identified by GWAS studies with low *p*-value thresholds, generally below 10*e*−8.

An interesting remark from the intersection analysis of the top 20 pipeline models is that sampling methods do not seem to affect classification methods that ultimately decide which SNPs are relevant to the model or not. Table 10 shows the unique combination of FS + Classifier that emerge from the top 20 pipeline models. We observe that in 3 out 8 cases the relevant features coincide for the FS + Classification configuration pipelines.

**Table 10:** Intersection of relevant features from top 20 pipeline models that coincide with the same configuration of FS + Classifier

FS + Classifier	# of relevant features selected by pipelines	# of features that match
ANOVA + LINEAR SVM	193 / 193 / 193	193
ANOVA + RF	144 / 193 / 193	144
ANOVA + KNN	95	N/A
RFE-LR + LINEAR SVM	148 / 148 / 148	148
RFE-LR + RF	257 / 238 / 305	3
RFE-LR + KNN	148 / 148	148
RLR-L1 + RF	3 / 7	3
RLR-L1 + KNN	13 / 17 / 16	12

### Functional SNP analysis

From 1224 unique variants identified in the 20 top pipelines, 1159 with reported rs signature were explored with Regulomedb (see Additional file 3). Eight SNPs showed a regulomedb score of “1f”, indicating that they are likely to affect binding protein and linked to expression of a gene target. Three out eight SNPs show a cis-effect expression in lung tissues, two SNPs at MAE (Macrophage Erythroblast Attacher also known as Human Lung Cancer Oncogene 10 Protein) *rs13147602* (*p*-values eQTLs =2.3*e*−5 and 6.9*e*−7, m-values = 1 and 1), *rs9424303* (*p*-value eQTLs =3.2*e*−24, m-value = 1) and one in *CEP104* (Centrosomal Protein 104), *rs6702916* (*p*-value eQTLs =6.9*e*−22, m-value = 1). Furthermore, three variants are likely to affect protein binding one at *PRKCZ* (Protein Kinase C Zeta) (*rs262669*), and two at *ADRB2* (Adenosine deaminase, RNA-specific, B2) (*rs4880878* and *rs10903495*). The former is likely to affect the *RUNX3* protein, a candidate tumor suppressor in many human tumors such as NSCLC [71] and *SPI1*, a transcriptor factor that may be related to NSCLC [72]. The second is likely to affect the *CTCF* protein, which regulates the *TERT* gene and its over-expression is important in lung cancer [73].

## Discussion and conclusions

The problem of missing heritability has been the focus of research and interest for many biologists and geneticists over several past years. With the coming age of the GWAS approach, the hope of identifying many genes involved in complex diseases arose. Indeed, many of these studies, applied to large case-control groups, have identified hundreds of genetic variants associated with complex diseases. However, the effect of most of these is too small in order to explain the risk or to make a valuable prediction, still holding many doubts about their use.

In this study we propose an alternative to the GWAS approach, based on a machine learning framework to analyze large-scale genetic data of complex diseases, identify relevant variants and perform patient stratification. We define this framework in a pharmacogenomics study in NSCLC patients subjected to first-line platinum-based treatment using a genome-wide imputed data of millions of SNPs.

After applying the 36 different experiments of the pipeline design, we found that the standard deviations of the CV F1 scores had low values, with std below 0.1 for more than a half of the models. This feature is important because it shows that the model selection process applied using CV is robust and suggest that the *CV F1* scores obtained in each experiment are close to the true values. This is also a sign that the final models, regardless of their performance, are stable and trustworthy because all of the steps from the pipelines were performed inside the k-fold CV loop. Not doing the latter is a common pitfall [74] in the application of ML methods. The main error is to apply “pre-processing steps” (missing value management, variance filter and standardization) and even feature selection and sampling techniques to the whole data-set upfront, before splitting into training and test data-sets, and only applying the CV to the classification model with the pre-filtered data.

Another characteristic of the experiments performed was that the *Test F1* scores were very close to their *CV F1* counterpart, almost 70% of them had differences below 0.05. This is important because suggests that the final models do not over-fit the data and are able to generalize and perform similarly on new unseen data.

The *F1*, *Precision* and *Recall* scores very much depend on the classification problem. For example, in [9, 75, 76] we can see similar accuracies and low *Recall* values for several algorithms. The performances (accuracies) obtained are very much in line with what has been reported in these articles. In our case, our best *F1* score is 0.72, which is considered to be acceptable for the problem at hand and the amount of data available.

The general criterion for classifying individuals with the machine learning framework was to focus on the models with the highest *CV F1* and *Test* scores. Specifically the class 0 *Recall* (*Test Recall (0)*), to keep track of low *FN* values. Remember we hope to obtain models with low *FN* values in order to avoid predicting that a certain patient responds to treatment, when in reality he/she does not.

We identified 1224 SNPs as the most relevant key features from the top 20 pipeline models (*CV F1* score >= 0.65). We believe that considering the rest of experiments with possible relevant functional variants are not appropriate for patient stratification because their *CV F1* are close to or smaller than 0.50. It is worth to mention that most of the identified variants were under genome-wide significance and have not been reported (*p*-value <10*e*−6) previously in the GWAS Catalog. Furthermore, only few of these variants are scored with a higher regulome score, having putative functional role as eQTLs in lung tissues or affecting binding proteins involved in well known lung cancer genes as *RUNX3*, *SPI1* and *CTCF*.

This study has the several limitations. Despite we obtained good classification measures, the sample size and therefore the size of the training data-set was small. We are aware that when applying the ML framework design, performing the “partial analysis” with the training and stability data-sets and later a separate “final analysis” with part of that same training data-set, introduces bias to the obtained results. We are also aware that the lack of an additional/independent sample to train and test the models is a limitation to stress the scores and the key features obtained. Given the difference in performance between the *Train F1* and *CV F1* scores (mean value of the differences equals 0.2), we believe there is room for improvement when the different models are trained with a larger data-set.

From our study, the machine learning approach is anticipated as an state-of-the-art, scalable and flexible methodology alternative to the classical GWAS analysis. Despite none of the SNPs identified as relevant by the combination of ML methods applied in this study were previously reported in the GWAS catalog (thresholds below 10*e*−6), we obtained a robust classification model using large-scale genomic data, that enlighten new involved genes. The effect results of these variants can be explained by the recently proposed the omnigenic model hypothesis, which states that complex traits can be influenced mostly by genes outside not only by the “core genes”, mainly found by the genome-wide significant SNPs, but also by the rest of genes outside of the “core pathways” with apparent unrelated biological functionality [77].

## Additional files

Additional file 1 General and class specific metrics of all 36 possible instantiations of pipeline using LC data-set. They are ordered by *CV F1*. (CSV 9 kb)

Additional file 2 Detailed description of evaluation metrics used in our experiments. Description of columns in Table 8. (DOCX 6 kb)

Additional file 3 1159 key features explored with Regulomedb database that are identified with the top 20 pipelines ranked by *CV F1* score. (CSV 31 kb)
