# Supplementary material for: Pipeline design to identify key features and classify the chemotherapy response on lung cancer patients using large-scale genetic data
Source: BMC Syst Biol. 2018 Nov 20;12(Suppl 5):97. doi: 10.1186/s12918-018-0615-5 (PMC6245589; doi:10.1186/s12918-018-0615-5)
Supplement: Supplementary file 2 — Detailed description of evaluation metrics used in our experiments. Description of columns in Table 8. (DOCX 6 kb) [file 12918_2018_615_MOESM2_ESM.docx]

Model Evaluation:

Some popular metrics used in imbalanced domains that consider the user preferences and, thus, take into account the data distribution are *Precision* (*P*) also known as *Positive Predictive Value* (*PPV*), *Negative Predictive Value* (*NPV*), *Recall* (*R*) also known as *True Positive Rate* (*TPR*) or *Sensitivity* and *True Negative Rate* (*TNR*) also known as *Specificity*, defined as follows [2] [1]:

$P=\frac{TP}{TP+FP}$ Eq. (1)

$NPV=\frac{TN}{TN+FN}$ Eq. (2)

$R=\frac{TP}{TP+FN}$ Eq. (3)

$TNR=\frac{TN}{TN+FP}$ Eq. (4)

In other words, the *Precision* corresponds to the proportion of examples classified as positive that are truly positive (Eq. (1)), the *Negative Predictive Value* corresponds to the proportion of examples classified as negative that are truly negative (Eq. (2)), the *Recall* corresponds to the proportion of truly positive examples that are classified as positive (Eq. (3)) and the *True Negative Rate* corresponds to the proportion of truly negative examples that are classified as negative (Eq. (4)) [3].

From the definition of the previous metrics we can see a clear relationship between *P* and *NPV* and between *R* and *TNR*. They each measure the same metric with respect to a specific class. Ee will refer to *P* as the *Precision of class 1* (*Precision (1)*), *NPV* as the *Precision of class 0* (*Precision (0)*), *R* as the *Recall of class 1* (*Recall (1)*) and *TNR* as *Recall of class 0* (*Recall (0)*).

Another popular and practical metric, that simultaneously measures the impact of several measures is the *F-measure* (F_β_). It combines the *Precision* and the *Recall* by a ratio specified by the *β* parameter [3].

$F_{\beta}=\frac{\left( 1+\beta\right)^{2}\cdot R\cdot P}{\left( \beta^{2}\cdot R \right)+P}$ Eq. (5)

In Eq. (5), if *β* = 1, then *Precision* and *Recall* are considered as being equally important. If *β* = 2, then *Recall* is considered to be twice as important as *Precision*. If *β* = 0.5, then *Precision* is considered to be twice as important as *Recall* [3]. In this study we'll use the *F-measure* with *β* = 1 denoted from now on as *F1*.

Using *Precision (1)*, *Recall (1)* and the F_β_ Eq. (5), we define the *F1 of class 1* (*F1 (1)*), and applying the same logic using the class 0 measures we obtain the *F1 of class 0* (*F1 (0)*).

We define the weighted version of any measure *M* as the average of the measure *M* for each class, weighted by the support, i.e. the number of true instances for each class [4]. Given the number of observations of class 1 and class 0, denoted as n_1_, n_0_ respectively, and using the previously defined measures we define the weighted versions of the *Precision*, *Recall* and *F1*:

$F1=\frac{\left( n_{1}\cdot F1\left( 1 \right) \right)+\left( n_{0}\cdot F1\left( 0 \right) \right)}{n_{1}+n_{0}}$ Eq. (6)

$Precision=\frac{\left( n_{1}\cdot Precision\left( 1 \right) \right)+\left( n_{0}\cdot Precision\left( 0 \right) \right)}{n_{1}+n_{0}}$ Eq. (7)

$Recall=\frac{\left( n_{1}\cdot Recall\left( 1 \right) \right)+\left( n_{0}\cdot Recall\left( 0 \right) \right)}{n_{1}+n_{0}}$ Eq. (8)

References:

[1] Paula Branco, Luis Torgo, and Rita Ribeiro. A survey of predictive modelling under imbalanced distributions. arXiv preprint arXiv:1505.01658, 2015

[2] Verónica Bolón-Canedo, Noelia Sánchez-Maroño, Amparo Alonso-Betanzos, José Manuel Benítez, and Francisco Herrera. A review of microarray datasets and applied feature selection methods. Information Sciences, 282:111-135, 2014.

[3] Andrew Estabrooks and Nathalie Japkowicz. A mixture-of-experts framework for learning from imbalanced data sets. In International Symposium on Intelligent Data Analysis, pages 34–43. Springer, 2001.

[4] Wikipedia. Weighted arithmetic mean, 2017. URL https://en.wikipedia.org/wiki/Weighted_arithmetic_mean.
